# Impact of class II and class III skeletal malocclusion on pharyngeal airway dimensions: A systematic literature review and meta-analysis

**DOI:** 10.1016/j.heliyon.2024.e27284

**Published:** 2024-03-05

**Authors:** Jensyll Rodrigues, Emmanouil Evangelopoulos, Ioannis Anagnostopoulos, Nisheta Sachdev, Ahmad Ismail, Rani Samsudin, Khaled Khalaf, Snigdha Pattanaik, Shishir Ram Shetty

**Affiliations:** a College of Dental Medicine, University of Sharjah, Sharjah, United Arab Emirates; b School of Dentistry, DY Patil University, India; c Institute of Dentistry, University of Aberdeen, United Kingdom

**Keywords:** Pharyngeal airway, Skeletal malocclusion, Cephalometric

## Abstract

**Background:**

This study is a pioneer systematic review and meta-analysis aimed at comparing the influence of Class II and Class III skeletal malocclusions on pharyngeal airway dimensions. It stands as the inaugural comprehensive assessment to collate and analyze the disparate findings from previously published articles on this topic. The objective of this study was to identify published articles that compare the effects of class II and class III skeletal malocclusion on the pharyngeal airway dimensions.

**Methods:**

An all-inclusive search for existing published studies was done to identify peer-reviewed scholarly articles that compared the influence of class II and class III skeletal malocclusion on pharyngeal airway dimensions. The search was done via five electronic databases: Cochrane Library, EMBASE, Scopus, Web of Science, and PubMed. Screening of the articles was done and the eligible studies were critically assessed using the Joanna Briggs Institute (JBI) Critical Appraisal Checklist.

**Results:**

The initial search yielded 476 potential articles of which, nine were finally included in this study for a total of 866 patients. Three studies were of cross-sectional design and six were of retrospective study design. Following a critical analysis and review of the studies, class III skeletal malocclusion had significantly larger volume and area measurements compared to class II skeletal malocclusion.

**Conclusion:**

Research in the field of literature has established that variations in skeletal classifications have a discernible effect on the size of the pharyngeal airways. With the advancement of skeletal malocclusions to a class III, there is an observed increase in both the volume and cross-sectional area of the airways.

## 1 Introduction

Respiratory function has been extensively studied about craniofacial growth and has consequently played a significant role in orthodontic diagnosis and treatment planning [[1], [2], [3]]. The Skeletal system seems to respond to the influences of adjoining tissues, as demonstrated by the functional theory of Moss. In particular, the mandible and cervical vertebrae grow because of functional relationships formed by all soft tissues and spaces operating in association with these bones [4]. The close anatomical relationship between the mandible, cervical vertebrae, and pharyngeal airway has aroused the interest of different research groups who have examined their relationships and demonstrated a correlation between skeletal malocclusion, head posture, and pharyngeal airway space [[5], [6], [7]].

The asymmetry of the face is described as an unproportional dimension, shape, and location of the jaws. This imbalance affects the appearance of the subjects which could impact social and psychological aspects considering the quality of life [8]. Dental development in humans is associated with malocclusions and dental defects at various developmental stages [9]. Management of these deformations calls for the prediction of the skeletal growth pattern to aid in the diagnosis and correction of these conditions. This integrates accurate estimation of the craniofacial growth patterns [10]. It is worth noting that malocclusions are influenced by the genetic makeup of individuals [9]. In addition, environmental and functional factors also contribute to the different classes of malocclusions [10]. It is essential to describe patterns for efficient communication in the orthodontic field. This called for the classification of cranio-maxillofacial patterns where skeletal classifications namely: class I, class II, and class III malocclusions came up. Emphasis is put on research on deformities which calls for dentists to have adequate knowledge about the management of the malocclusions to reduce the adversity of their effects. Characteristics of class III malocclusions are often evident at an early age requiring early management [11].

On the other hand, the pharyngeal airway is a volumetric structure that is influenced by different skeletal patterns [12]. The construction and dimensions of the airway are influenced by the anatomy of the adjacent structures that have a direct impact on the volume and craniofacial growth pattern [13]. The human pharynx is an important structure in the respiratory system and is located at the posterior side of the mouth and nasal cavity, above the esophagus. It is divided into; the nasopharynx, oropharynx, and laryngopharynx which forms the basis of the three classifications of the pharyngeal airways [14]. Among the different classifications of the airways, oropharyngeal and nasopharyngeal airways are separated by the retropalatal section of the maxilla while the oropharyngeal and the laryngopharyngeal airways are separated by epiglottis tip [15].

When the respiratory function changes, particularly during chronic mouth breathing, the positions of the head, neck, cervical vertebrae, jaws, and teeth are affected [1,[16], [17], [18]]. Ricketts defined this association as respiratory obstruction syndrome [2]. Others called it adenoid facies or long face syndrome [1,18,19]. Adenoid and tonsil hypertrophy, mouth breathing, narrow external nares, V-shaped maxillary arch, open bite, tongue thrusting, excessive anterior facial height, incompetent lip posture, protruding maxillary teeth with a Class II malocclusion, a steep mandibular plane, posterior dental crossbites, a forward inclined cervical column, and an extended head position are common traits in the orthodontic and medical literature associated with altered respiratory function [16].

Extensive research in the past has reported associations between respiratory obstruction and skeletal malocclusion [[18], [19], [20], [21], [22], [23]]. Linder-Aronson et al. proposed that children with severe nasal obstruction who underwent adenoidectomy had more forward mandibular growths than patients with clear pharyngeal airways who did not undergo surgery.

The upper airway has become an area of interest regarding its construct and dimensions due to its relationship with obstructive sleep apnea and is closely associated with different cranial morphologies [24]. Evidence shows that skeletal anomalies are a major hindrance to the flow of air in subjects [13].

Obstructive sleep apnea is a relatively common condition caused by recurrent airway obstruction during sleep. Airway occlusions can occur several hundred times during the night, resulting in sleep fragmentation and hypoxemia. It is frequently observed in obese patients. Patients with this condition can experience several symptoms, such as excessive daytime sleepiness, heart failure, systemic hypertension, and cardiac arrhythmias. Intellectual deterioration and sexual impotence have also been reported. During inspiration the intra-pharyngeal pressure becomes negative. However, airway collapse is prevented by the pharyngeal abductor and dilator muscles. These muscles are hypotonic during sleep but are active during daytime respiration. When this occurs, PAS stability becomes dependent on pharyngeal tissue compliance and size. Tissue compliance is a measure of the consistency or firmness of a tissue. A tissue that lacks compliance tends to be flaccid. Currently, little is known about pharyngeal tissue compliance. Interestingly, airway narrowing and sleep apnea were correlated with patients with severe mandibular retrognathia [25].

The oropharyngeal airway could be most affected by the size and location of the tongue [15]. Additionally, the location of the upper and lower jaws is associated with constriction of the anteroposterior aspect of the airway [13]. Upper airway obstruction and thus dysfunction leads to oral breathing which impacts craniofacial and skeletal morphology causing malocclusion [26]. Researchers have directed effort and attention towards cognizing the relationship between pharyngeal airway and dental anomalies. Different skeletal patterns have been explored [26].

This paper aims to compare class II and class III malocclusion with the primary objective of appraising the existing evidence of the relationship with the pharyngeal airways. Evidence from studies shows that class II malocclusion is predominant among patients who sought orthodontic care [27]. Surgical procedures are carried out to correct the anomalies with the primary objective of improving the appearance of patients besides improving the functional aspect of the occlusion [28]. In addition, surgery aims at restoring skeletal malocclusion and irregularities in the soft tissues surrounding the jaws. For instance, it is assumed that mandibular development improves the quality of sleep for patients with obstructive sleep apnea [29]. Advancement in the interventions depends on the understanding of the relation between the malocclusions and the pharyngeal airways.

According to the literature, functional appliances help the mandible reposition itself anteriorly. However, some researchers believe that the changes are associated with the dentoalveolar region and that the actual skeletal changes are minimal [30]. Nevertheless, several studies using functional appliances have observed a significant improvement in the pharyngeal airway volume after treatment with appliances compared with the volume before treatment [31,32].

Initially, pharyngeal airways were analyzed in two dimensions using radiographs. However, challenges were experienced like distortion of images among others. Lateral cephalography was also widely applied in evaluating characteristics of the airways regarding the sagittal plane [33]. Three-dimensional analysis and evaluation of the airways have been developed [34]. Studies of the airways depend on the 3-D scans used in the management of jaw discrepancies. Volumetric and areal comparison gives a clearer representation of the correlation between the airways and skeletal patterns. Advancements in cone beam computed tomography technology have introduced the possibility of rendering high-quality dentofacial images at a very precise level. Three-dimensional analysis allows the distinctive advantage of examining objects in a 1:1 ratio without the worries of distortion, magnification, or superimposed anatomical structures typical with 2-dimensional scans [35].

In the quest for knowledge on the relationship between different skeletal malocclusions and pharyngeal airways, techniques have been devised to analyze three-dimensionally the characteristics of the airways. Cone beam computed tomography is widely employed in the studies reviewed. It is applied in dental imaging due to its economical and low radiational impacts in the study of skeletal patterns [36]. This study reviews articles that employed analysis of images to compare volume and area besides other cephalometric variables in the understanding of the pharyngeal airways. The dimensions measured were used in the comparison of class II and class III skeletal malocclusion forming a basis for the relationship between the airways and different classes of skeletal malocclusions.

This paper compares volumetrically class II and class III skeletal malocclusion based on their impact on the dimensions of the pharyngeal airway by reviewing current published literature on the two classes of skeletal malocclusions. Other characteristics of the pharyngeal airways are also used in the comparison like; retroglossal and retropalatal compartment dimensions like their total areas and volume. These metrics form a conclusive criterion for analysis of the pharyngeal airways given that it is a volumetric structure directly impacted by the different skeletal classes in terms of its dimensions. Some studies further divided the evaluation of the airways into upper and lower sections which were considered relevant in the analysis and understanding of the impact of different skeletal classes on the dimensions of the pharyngeal airways. Additionally, this study aims at appraising scientific evidence of the relationship between skeletal malocclusions and the airways to better understand the subject and subsequently aid in the management of the adverse effects of dental anomalies as the etiology of malocclusion is multifactorial, airway study and evaluation should be an integral part of the initial diagnosis to help diagnose possible obstructive airway syndromes, better develop a final treatment plan, and understand possible risk factors for airway-related problems. Furthermore, this knowledge would help orthodontists collaborate with the obstructive sleep apnea team and fabricate a mandibular protruding device or prepare patients for orthognathic surgery to help with improved breathing [19]. Therefore, an assessment of the pharyngeal structure in association with the orthodontic and orthognathic surgery diagnosis, the treatment planning process, and the diagnosis and treatment of any airway-related dysfunction might be beneficial.

### 1.1 Objectives and research question

This paper is the pioneer systematic review and meta-analysis on the influence of class II and class III skeletal malocclusion on pharyngeal airway dimensions This study had primary objectives of (a) identifying existing published research papers comparing the influence of class II and class III skeletal malocclusion on pharyngeal airway dimensions, (b) exploring the evidence of the impact of class II and class III skeletal malocclusion on pharyngeal airway dimensions, and (c) performing a meta-analysis on the data thereby extracted from included studies.

### 1.2 Research question

What is the evidence of the impact of class II and III skeletal malocclusion on pharyngeal airway dimensions, and how do these malocclusions correlate with changes in the volume and area of the airways?

## 2 Methodology

This study was reported following the Preferred Reporting Items for Systematic Reviews and Meta-analyses (PRISMA) standards of quality for the planning, conducting, and reporting of systematic reviews and meta-analyses [37].

### 2.1 Eligibility criteria (inclusion/exclusion)

Only studies reported in the English language to date were included in the study. The included studies should have compared the pharyngeal airway dimensions of class II with class III skeletal malocclusion.

Reviews articles, meta-analyses, protocols, opinion pieces, pilot studies, feasibility, and studies with less than 8 participants. Studies that addressed the impact of surgical procedures on the correction of skeletal malocclusion were not included. Studies with unclear methodology or outcomes were also not included.

### 2.2 Search strategy

An inclusive search of the literature was conducted to find potential peer-reviewed scholarly articles that compared the effect of class II with class III skeletal malocclusion on pharyngeal airway dimensions. The search was run via five electronic databases: PubMed, Cochrane Library, EMBASE, Scopus, and Web of Science. Search terms were used in different combinations for the different databases, including the following keywords: “pharyngeal airway”, “oropharyngeal airway”, “nasopharyngeal airway”, “pharyngeal space”, airway, “pharyngeal airway space”, “Skeletal malocclusion”, “dental anomaly”, “dental malocclusion”, “skeletal irregularity”, “skeletal malformation”, “skeletal jaw discrepancy".

### 2.3 Review methods

#### 2.3.1 Data selection and extraction

The last date for the literature search was search was December 6th, 2022. Two examiners (J.R and S.S) separately reviewed the articles for eligibility based on their titles and abstracts provided in the database.

Later, the 2 examiners selected full-text articles for the systematic review based on the inclusion criteria. In case of disagreement, a third examiner (S.P) would provide input for finalizing the decision. A search strategy was carefully formulated and a comprehensive search for peer-reviewed scholarly articles was conducted. There was no automatic removal of articles by the citation manager. Duplicates were removed and the remaining potential articles were screened by abstract and titles to check eligibility in line with the specified inclusion and exclusion criteria. This was followed by full-text screening after retrieval of the articles. The included articles were carefully selected after critical analysis to check if they met the inclusion criteria favorable for this study. Data was methodically extracted and evaluated for accuracy. The extracted data were summarized in the study descriptor table. Table 1 includes the author, study design, sample size, mean or median age, technique, the purpose of the study, outcomes, and main findings of the individual studies.

**Table 1:** Study Descriptor table.

Author	Study Design	Sample Size	Mean Age	Technique	Purpose of study	Outcomes	Conclusions
(Alves et al., 2008) [19]	Cross-sectional Study	60	17.75	computed tomography scans	To investigate upper airway space in normal nasal breathing patients having skeletal pattern of classes II and III.	Retroglossal cross-section area mean in retrognathic cases was found larger in male than in female subgroup and total volume and area means were larger in male subgroup in prognathic cases.	Evaluation of upper airway space should be integrated in the diagnosis and treatment planning to achieve functional balance and consistency of the results.
(Castro-Silva et al., 2015) [20]	Retrospective Study	60	29.5	Cone-Beam Computed Tomography Analysis	To make a 3-D evaluation of the pharyngeal airway space in patients with Class I, II and III malocclusions.	The mean volume and area for class III patients were statistically bigger than for classes I and II	Class III patients presented a bigger PAS, when compared to classes I and II patients.
(Chokotiya et al., 2018) [21]	Retrospective Study	120	15.54	Cephalometric radiography	To evaluate how pharyngeal airway dimensions were affected by different skeletal malocclusions.	In pharyngeal measurements, the difference is significant only in the Class II and males have statistically significant higher mean values than the females	The sagittal skeletal pattern does not seem to influence the variations in the upper airway dimension
(Claudino et al., 2013) [22]	Retrospective Study	54	11.68	Cone-beam CT (CBCT) scans	To characterize the volume and the morphology of the pharyngeal airway in adolescent subjects, regarding their facial skeletal pattern	The minimum areas in the Class II group were significantly smaller than in Class III group for the lower pharyngeal portion, the velopharynx, and the oropharynx, respectively, and significantly smaller than the Class I group for the velopharynx	A negative correlation was observed between the ANB value and airway volume in the lower pharyngeal portion and the velopharynx
(Dogan et al., 2020) [23]	Retrospective Study	45	16.77	cone beam computed tomography (CBCT)	To compare pharyngeal airway dimensions (PADs) between orthodontic malocclusions	PADs at (A/G)-B and (A/G)-Pog decreased in Class II, whereas PAD at (A/G)-A decreased in Class III, as compared to control	The upper and lower PADs are affected by different dental and skeletal malocclusions
(Gholinia et al., 2019) [24]	Cross-sectional Study	105	Unspecified	Analysis using AutoCAD software	To evaluate upper airway in different skeletal classifications of jaws in lateral cephalogram and its relation to age and gender.	Hypo pharyngeal airway depth, the nasopharyngeal airway depth and soft palate space were significantly different in skeletal classes	Upper airway dimension varies in different skeletal classes, developmental ages, and gender
(Jayaratne & Zwahlen, 2016) [25]	Retrospective Study	62	Unspecified	Cone-beam CT (CBCT) scans	To compare the anthropometric dimensions of the oropharyngeal airway in skeletal class II and III deformity patients	The average surface area, most constricted area, and anterior-posterior (AP) length of this constriction of both the RG and RP compartments were significantly larger in the class III deformity group	There were significant differences in morphological characteristics of the oropharyngeal airway in individuals with skeletal class II and III deformities
(Nath et al., 2019) [26]	Retrospective Study	180	Unspecified	Cone-beam CT (CBCT) analysis	To explore the influence of skeletal malocclusion on the oropharyngeal airway volume and the difference in the airway volume among gender and the different types of skeletal malocclusion	There was a significant result regarding oropharyngeal airway volume among different types of skeletal malocclusion	A maxillofacial radiologist is importantly placed in the assessment of the retropalatal and retroglossal volume of the oropharyngeal airway.
(Ünal & Soydinç, 2021) [27]	Cross-sectional Study	180	Unspecified	Cone-Beam Computed Tomography Analysis	To evaluate the relationship between pharyngeal airway and craniofacial structures by 3-D imaging methods in individuals with different skeletal anomalies	No statistically significant difference in volumetric measurements between Class 1, Class 2 and Class 3 groups	Malformations in the sagittal direction and different facial types in the vertical dimension may be related to pharyngeal airway volume and dimension

#### 2.3.2 Methodological quality assessment

The Joanna Briggs Institute (JBI) Critical Appraisal Checklist for analytical cross-sectional study (last amended in 2017) was used to methodologically assess the quality of the included studies [38]. The results of the assessment are shown in Table 2. The GRADE evidence profile by (Zhang et al., 2018) was used to assess the strength of the evidence [39] Table 3.

**Table 2:** Results of methodological quality assessment.

Study	Clear definition of inclusion criteria	A clear description of study subjects and setting	Measurement of exposure	Standard criteria for measuring condition	Identified confounding factors	Strategies to counter confounding factors	Valid and reliable measurement of outcomes	Appropriate statistical analysis	Overall appraisal
(Alves et al., 2008) [20]	Unclear	Yes	Not applicable	Yes	Yes	Unclear	Yes	Yes	Include
(Castro-Silva et al., 2015) [21]	Yes	Yes	Not applicable	Yes	No	Not applicable	Yes	Yes	Include
(Chokotiya et al., 2018) [22]	No	Yes	Not applicable	Yes	Yes	Unclear	Yes	Yes	Include
(Claudino et al., 2013) [23]	Yes	Unclear	Not applicable	Yes	No	Not applicable	Yes	Yes	Include
(Dogan et al., 2020) [24]	Yes	No	Not applicable	Yes	Yes	Unclear	Yes	Yes	Include
(Gholinia et al., 2019) [25]	Yes	Unclear	Not applicable	Yes	No	Not applicable	Yes	Yes	Include
(Jayaratne & Zwahlen, 2016) [26]	Unclear	Yes	Not applicable	Yes	Yes	Yes	Yes	Yes	Include
(Nath et al., 2019) [27]	No	Unclear	Not applicable	Yes	No	Not applicable	Yes	No	Include
(Ünal & Soydinç, 2021) [28]	Yes	No	not applicable	Yes	No	Not applicable	Yes	Yes	Include

**Table 3:** GRADE evidence profile (Zhang et al., 2018).

	Quality Assessment							Number of Patients		Quality	Importance
Outcome	Number of Studies	Study Design	Risk of Bias	Inconsistency	Indirectness	Imprecision	Other Considerations	Class II	Class III		
Total Airway Volume	2	Cross sectional and retrospective study	Low Shown by quality assesment results	Not serious Shown by the forest plot	Not serious	Not serious Shown by the forest plot	None	80	80	Moderate	Critical
Retroglossal Volume	2	Cross sectional and retrospective study	Low Shown by quality assesment results	Not serious Shown by the forest plot	Not serious	Not serious Shown by the forest plot	None	57	65	Moderate	Critical
Retropalatal volume	2	Cross sectional and retrospective study	Low Shown by quality assesment results	Not serious Shown by the forest plot	Not serious	Not serious Shown by the forest plot	None	57	65	Moderate	Critical

## 3 Results

### 3.1 Search results

**Fig. 1:**
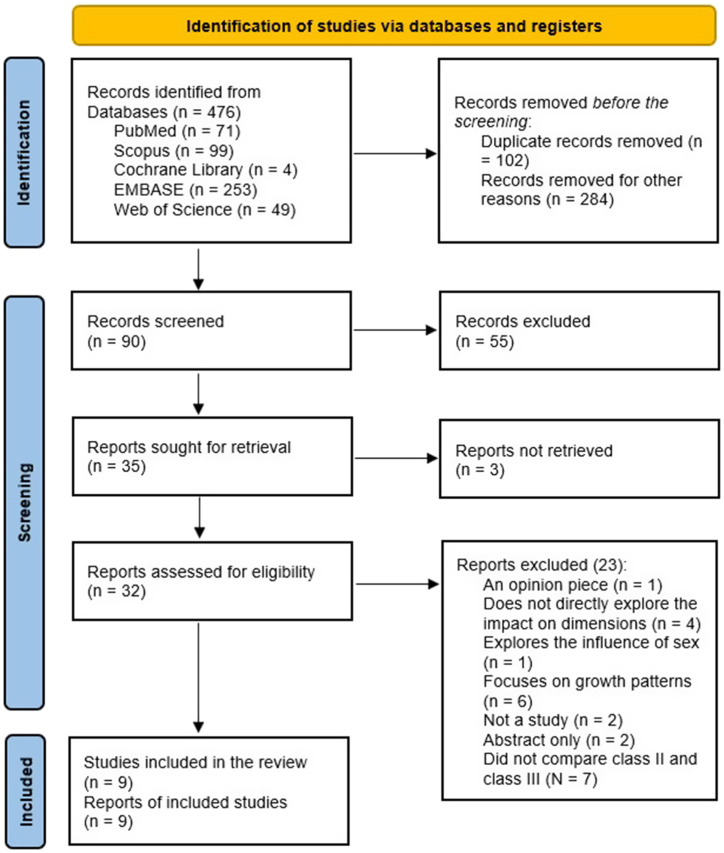
PRISMA flow diagram for the results of identification and screening of articles.

Database search produced 476 articles out of which 102 duplicate records were removed. The titles and abstract screening conducted resulted in the elimination of 284 journals. Articles that were thereafter screened were 90. A further 55 articles were excluded based on exclusion criteria. Thirty-five articles were sought for retrieval. Three of which could not be retrieved. Thirty-two articles were further assessed for eligibility. One opinion piece was eliminated and four others that did not directly explore the impact on the pharyngeal airway dimensions were excluded. One study that discussed the influence of sex on airway dimensions was also excluded. Six other articles were excluded as they only focused on the growth patterns rather than the impact of class II and class III skeletal malocclusion on the dimensions of the airway. Two opinion pieces were excluded. Two other articles had abstracts only and seven others that did not compare class II and class III skeletal classifications were excluded. This screening finally yielded nine articles that were eventually included in this paper and were considered eligible for review as shown in Fig. 1.

### 3.2 Results of individual studies

This study included three cross-sectional design studies and six retrospective studies for a total of 866 patients. Dogan et al. (2020) [40] had the smallest sample size of 45 patients whereas Nath et al. (2019) [41] and Ünal & Soydinç, (2021) [42] had the largest sample sizes of 180 patients. The nine studies explored volumetric evaluation of the pharyngeal airways and their correlation with skeletal classes.

### 3.3 Total airway volume

**Fig. 2:**
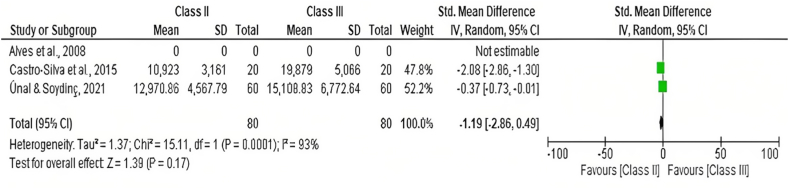
Forest plot comparing total pharyngeal airway volume among class II and class III patients.

Two studies that are; Castro-Silva et al. (2015) [43] and Ünal & Soydinç, (2021) [42] reported quantitively on the total airway volume in patients with class II and class III patients. The data was analyzed using the review manager while applying the random effects model. The total airway volume was compared between class II and class III skeletal malocclusions as shown in Fig. 2. The forest plot shown in Fig. 2 favors class II skeletal malocclusion having a smaller total airway volume even though the standard mean difference is small compared to class III.

### 3.4 Retroglossal volume

**Fig. 3:**
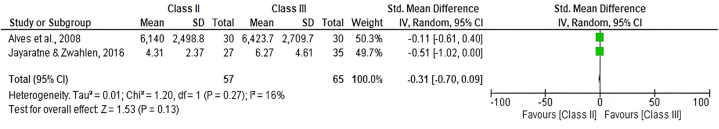
Forest plot comparing retroglossal volume in the upper airway among class II and class III patients.

Two studies; Alves et al. (2008) [44] and Jayaratne & Zwahlen, (2016) [45] reported quantitative data which was analyzed using the review manager regarding retroglossal volume where the random effects model was applied. The retroglossal volume was smaller in class II skeletal malocclusion as shown in Fig. 3. Different skeletal classifications have an impact on the retroglossal volume progressively enlarging from class II to class III. The forest plot shown in the figure favors class II skeletal malocclusion having smaller retroglossal volume even though the standard mean difference is small compared to class III.

### 3.5 Retropalatal volume

**Fig. 4:**
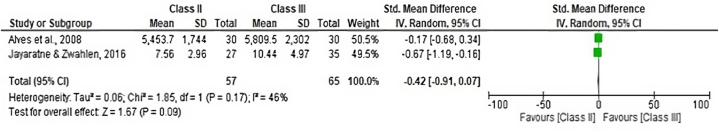
Forest plot comparing retropalatal volume in the upper airway among class II and class III patients.

Alves et al. (2008) [44] and Jayaratne & Zwahlen, (2016) [45] reported quantitative data which was analyzed using the review manager regarding retropalatal volume where the random effects model was applied. The retropalatal volume was smaller in class II skeletal malocclusion as shown in Fig. 4. Different skeletal classifications have an impact on the retropalatal volume progressively enlarging from class II to class III. The forest plot shown in the figure favors class II skeletal malocclusion having smaller retropalatal volume even though the standard mean difference is small compared to class III.

## 4 Discussion

The findings of this review appraise the already present vast scientific research, drawing conclusions between skeletal malocclusion and airway, where wider airways are observed in skeletal class III whereas narrower airways are in skeletal class II. Scientific evidence shows a correlation between pharyngeal airways and adjacent skeletal structures besides soft tissues. Additionally, the airways play a vital role in respiration [46]. Intermittent reductions in respiratory function which is primarily caused by constriction of the upper airway are associated with obstructive sleep apnea. Highlighted risk factors for the condition include the occurrences that limit the dimensions of the airways in which the different skeletal classes play a significant role [47]. This adds to the vital nature of the relationship between different skeletal classes and airway volume.

This paper aimed to appraise scientific evidence of this relationship by finding published literature comparing three-dimensionally the correlation between class II and class III malocclusions. Patients with class II and class III skeletal malocclusions were included in this study. This is the first systematic literature review and meta-analysis on the comparison of the impact of class II and class III skeletal malocclusions exploring cephalometric measurements as a basis of comparison. Total airway volume was measured by three included studies besides total area. Other dimensions like minimum constricted area and upper airway dimensions among other dimensions were also reported. Great interest and attention from orthodontists have been reported by research [26].

Evaluation of the pharyngeal airways has advanced with technology into three-dimensional analysis from the traditional lateral cephalograms which were two-dimensional. It is worth noting that cone beam computed tomography has been widely applied considering the relationship between the risks and benefits that it offers particularly being economical and having low radiation [48]. Parameters are evaluated from the analysis of scans from cone beam computed tomography as reported by the studies included in this paper. Three-dimensional analysis was carried out, and measurements were derived. In analyzing pharyngeal airway volume, segmentation is done for better visualization [46].

Total volume was reported by Castro-Silva et al. (2015) [43] and Ünal & Soydinç (2021) [42] to be larger in class III compared to class II skeletal malocclusion. The total airway volume encompasses the upper and lower airway dimensions. Ünal & Soydinç, (2021) [42] reported nasopharyngeal airway volume which was greater among class III patients compared to class II. Jayaratne & Zwahlen, (2016) [45] pointed out from a three-dimensional morphometric analysis that oropharyngeal airway volume was significantly greater in skeletal class III patients (16.7 ± 9.04 mm^3^) compared to skeletal class II patients (11.87 ± 4.01 mm^3^).

Evidence of the impact of malocclusions on the retroglossal and retropalatal volume and area has also been reported showing a statistical significance between skeletal class II and class III patients. Jayaratne & Zwahlen, (2016) [45] reported that the average surface area of the retropalatal and retroglossal sections was considerably greater in class III skeletal malocclusion patients. The dimensions of the retropalatal section were also reported independently by Jayaratne & Zwahlen, (2016) [45] and Alves et al. (2008) [44] regarding the length, volume, and average cross-section area to be larger in skeletal class III patients. It was also noted to be greater than the retroglossal section in both the discrepancy classes.

A significant relationship between the pharyngeal airway and skeletal classes was also appraised regarding the minimum constricted area as reported by Jayaratne & Zwahlen (2016) [45] and Claudino et al. (2013) [49]. There was no statistically significant variation of the most constricted area between the class II and class III skeletal malocclusion. However, the anteroposterior length of the constriction was notably larger in class III in both retroglossal and retropalatal sections [45]. C. Maspero et al. [50] also concluded their study that the sagittal dimension on the posterior oropharyngeal airway and length of the soft palate was increased after the correction of mandibular retrusion in patients with a class II malocclusion.

A dominant comparison criterion among the studies reviewed in this paper was based on gender differences and their impact on the pharyngeal airway dimensions. Chokotiya et al. (2018) [51], Claudino et al. (2013) [49], and Gholinia et al. (2019) [52] reported consistently that males have larger retroglossal volume, minimum and average cross-sectional compared to females with the malocclusions. Dogan et al. (2020) [40] reported a volumetric evaluation of the nasopharynx, oropharynx, hypopharynx, and lower pharynx the length of the soft palate regarding the mean values and standard deviations. However, there was no significant statistical variation between the class II and class III malocclusions. Conversely, Claudino et al. (2013) [49] reported that there was no significant association between airway volume and skeletal pattern regarding the nasopharynx and hypopharynx in the upper pharyngeal section.

Evaluation of the pharyngeal airways as reported by Nath et al. (2019) [41] was done considering the ANB angle which had an inverse correlation with the retropalatal cross-section area while the SNB angle had a direct correlation with the retropalatal cross-section area. On the other hand, the ANB angle had an inverse correlation with retroglossal while the SNB angle had a direct correlation with retroglossal cross-section area. This review appraises the scientific evidence of the impact of different skeletal classes on pharyngeal airway dimensions while comparing class II with class III malocclusions.

### 4.1 Study limitations

A control group could not be included as reported by some studies considering the ethical issues of subjecting participants without deformities to radiation. Methodologic limitations regarding the method of assessment and unavailable longitudinal studies evaluating the airways, and complex causes of discrepancy. Patients referred for orthognathic surgery are more likely to have severe oropharyngeal discrepancy due to the skeletal structure compared to those referred for orthodontic treatment only.

### 4.2 Clinical implications

Airways are an important anatomic structure for orthodontists who evaluate the maxillofacial region and perform osteotomies. Differences in the singular spot associated with a skeletal class are discussed in this paper. In addition, the impact of different skeletal classes on the pharyngeal airway dimensions can significantly contribute to the planning of orthodontic treatment. Stimulation of sagittal growth of the mandibles, maxillary palatal expansion, and protraction may potentially be useful in enlarging the pharyngeal airway thereby reducing the adverse effects of obstructive sleep apnea. Orthodontic treatment is a possible intervention method for sleep-disordered respiration. Independent anthropometric analysis of retroglossal and retropalatal sections may be vital in the planning of orthognathic surgery. This study appraises the techniques used in the evaluation of the pharyngeal airways which helps in providing essential information that was previously limited while using the traditional two-dimensional cephalograms.

## 5 Conclusion

As the body of scientific knowledge expands, highlighting the correlation between the volume and area of pharyngeal airways and various skeletal classes, this research substantiates the influence that class II and class III malocclusions have on airway size. The study observed that class II malocclusions present a statistically notable difference in airway dimensions when compared to the considerably larger dimensions found in class III malocclusions. Furthermore, the study acknowledges a gender-based disparity in airway size, with males exhibiting significantly greater dimensions. The outcomes of this review underscore the necessity for additional research into the effects of different skeletal classes on pharyngeal airway dimensions, given the scarcity and dated nature of existing studies. Such research is instrumental in enhancing orthodontic and surgical interventions, aiming to counteract the negative outcomes of malocclusions, especially as technological advancements continue to evolve and the imperative to maintain current scientific evidence grows.

Nonetheless, there is a pressing need for long-term clinical follow-up studies and further research to determine the enduring effects of orthodontic and surgical treatments on the stability of airway dimensions. Prospective research should also consider the genuine necessity for such interventions and establish a comprehensive risk-benefit analysis for these procedures.

## Data availability statement

Data available at Figshare.com 10.6084/m9.figshare.25283131.

## CRediT authorship contribution statement

**Jensyll Rodrigues:** Writing – review & editing, Writing – original draft, Visualization, Validation, Supervision, Software, Resources, Project administration, Methodology, Investigation, Funding acquisition, Formal analysis, Data curation, Conceptualization. **Emmanouil Evangelopoulos:** Writing – review & editing, Investigation, Funding acquisition, Formal analysis, Data curation, Conceptualization. **Ioannis Anagnostopoulos:** Writing – review & editing, Investigation, Funding acquisition, Formal analysis, Data curation, Conceptualization. **Nisheta Sachdev:** Writing – review & editing, Investigation, Funding acquisition, Formal analysis, Data curation, Conceptualization. **Ahmad Ismail:** Writing – review & editing, Investigation, Funding acquisition, Formal analysis, Data curation, Conceptualization. **Rani Samsudin:** Writing – review & editing, Investigation, Funding acquisition, Formal analysis, Data curation, Conceptualization. **Khaled Khalaf:** Writing – review & editing, Investigation, Funding acquisition, Formal analysis, Data curation, Conceptualization. **Snigdha Pattanaik:** Writing – review & editing, Investigation, Funding acquisition, Formal analysis, Data curation, Conceptualization. **Shishir Ram Shetty:** Writing – review & editing, Writing – original draft, Visualization, Validation, Supervision, Software, Resources, Project administration, Methodology, Investigation, Funding acquisition, Formal analysis, Data curation, Conceptualization.

## Declaration of competing interest

The authors declare that they have no known competing financial interests or personal relationships that could have appeared to influence the work reported in this paper.
